# Posttraining survey of recent pediatric gastroenterology fellowship graduates

**DOI:** 10.1002/jpr3.70050

**Published:** 2025-06-24

**Authors:** Christopher J. Moran, Christine K. Lee, Niviann Blondet, Rula Harb, Galen S. Hartman, Michael Herzlinger, Candi Jump, Priya S. Rolfes, Aliza Solomon, Arvind Srinath, Cary G. Sauer, Sarah S. Lusman, Daniel Mallon

**Affiliations:** ^1^ Division of Pediatric Gastroenterology, Hepatology, and Nutrition, Pediatric Gastroenterology Mass General for Children Boston Massachusetts USA; ^2^ Division of Pediatric Gastroenterology, Hepatology and Nutrition Boston Children's Hospital Boston Massachusetts USA; ^3^ Pediatric Gastroenterology Seattle Children's Hospital Seattle Washington USA; ^4^ Pediatric Gastroenterology, Hepatology and Nutrition Children's Hospital Los Angeles Los Angeles California USA; ^5^ Pediatric Gastroenterology SUNY Upstate Syracuse New York USA; ^6^ Pediatric Gastroenterology Warren Alpert Medical School of Brown University Providence Rhode Island USA; ^7^ Pediatric Gastroenterology Medical University of South Carolina Summerville South Carolina USA; ^8^ Pediatric Gastroenterology, Hepatology and Nutrition Children's Hospital Colorado Denver Colorado USA; ^9^ Pediatric Gastroenterology, Hepatology and Nutrition Weill Cornell Medicine New York New York USA; ^10^ Pediatric Gastroenterology University of Pittsburgh Medical Center Children's Hospital of Pittsburgh Pittsburgh Pennsylvania USA; ^11^ Pediatric Gastroenterology Emory University and Children's Healthcare of Atlanta Atlanta Georgia USA; ^12^ Division of Pediatric Gastroenterology, Hepatology and Nutrition Columbia University Irving Medical Center New York New York USA; ^13^ Division of Pediatric Gastroenterology, Hepatology, and Nutrition, Cincinnati Children's Hospital Medical Center University of Cincinnati College of Medicine Cincinnati Ohio USA

**Keywords:** graduate medical education, mentorship, procedural skills, training

## Abstract

**Objectives:**

The North American Society for Pediatric Gastroenterology, Hepatology, and Nutrition (NASPGHAN) Training Committee conducted a survey of recent fellowship graduates to assess their confidence in procedure performance, disease management, practice habits, and satisfaction with mentorship.

**Methods:**

The survey was developed by the Training Committee members and distributed during the summer of 2023 to fellowship graduates who finished training between 2018 and 2023. Confidence levels regarding treating specific diseases and performing gastrointestinal procedures were assessed, including analysis comparing the data to 2015 survey results.

**Results:**

The response rate was 21% (140/676). Confidence levels in the performance of most procedures and management of most diseases were high. Graduates of smaller programs reported greater confidence in performing percutaneous endoscopic gastrostomy placement and percutaneous liver biopsy. Outstanding research mentorship was reported more commonly with mentors funded via the National Institute of Health (NIH) than non‐R/K funded mentors (54% vs. 28%, *p* = 0.002). Outstanding clinical and career mentorship was similar between large‐sized, medium‐sized, and small‐sized programs. Preparedness for job hunting improved with time (52% vs. 30%, *p* = 0.005) while preparedness for advocacy work decreased (39% vs. 58%, *p* = 0.007).

**Conclusion:**

Respondents reported high confidence in many core activities of pediatric gastroenterology. Satisfaction with research mentorship was higher for NIH‐funded mentors. Confidence in performing certain procedures declined over time possibly because some centers shifted the responsibility of those procedures to other specialties. Improved confidence in some training‐related topics such as job‐hunting preparedness coincided with changes made to the curriculum for NASPGHAN's fellows conferences.

## Abstract (highlights)

**What is Known:**

- Training in pediatric gastroenterology requires the development of disease knowledge, procedural skills, and research skills.

**What is New:**

- Confidence among recent pediatric gastroenterology graduates is high in disease management and procedural skills despite most respondents reporting moderate to severe impact from COVID‐19 pandemic.
- Graduates report relatively low rates of confidence in patient advocacy and research skills.
- Graduates describe higher rates of confidence in the job hunt and contract negotiations since changes were made to the NASPGHAN 3rd Year Fellows Conference.

## 1 INTRODUCTION

Training in pediatric gastroenterology requires development of both cognitive skills related to medical knowledge and clinical decision making as well as technical skills for performing diagnostic and therapeutic procedures. Although exposure to a high volume of cases during training is important, entrustable professional activities (EPAs) focus on the acquisition of skills to independently care for patients and perform procedures rather than accrual of high case volumes.^1^ Some hospitals have begun to transition some procedures historically performed by pediatric gastroenterologists (such as percutaneous liver biopsy and percutaneous endoscopic gastrostomy [PEG] tube placement) to other subspecialists. The lack of a broad self‐assessment of this trend does limit strategies for program improvement.

Complicating this already complex need to acquire skills is the fact that recent graduates of pediatric gastroenterology training programs experienced their education during the height of the COVID‐19 pandemic. Many pediatric gastroenterology trainees reported substantial changes during their training such as reduced endoscopic procedure volume, altered inpatient and outpatient volumes, growth of telehealth, and pivot to virtual educational sessions due to stay‐at‐home orders.^2^ Among the many parameters of pediatric gastroenterology training, fellows cited procedure training as having been impacted the most with >50% of fellows during the 2020–2021 academic year reporting a major effect on their procedural experience.^3^ The longer‐term impact that COVID‐19 pandemic had on cumulative training experience has not been fully assessed.

The Training Committee for the North American Society for Pediatric Gastroenterology, Hepatology, and Nutrition (NASPGHAN) conducted this survey of recent graduates to assess confidence levels of trainees in managing various gastrointestinal (GI) diseases and performing procedures and to assess the overall effect that the COVID‐19 pandemic had on fellowship training.

## 2 METHODS

The survey tool was developed by the NASPGHAN Training Committee subgroup with a specific focus on understanding the confidence levels regarding common GI procedures, disease processes, and professional habits of graduating pediatric gastroenterology fellows. Once the survey was finalized, cognitive interviewing was conducted with representative participants to ensure clarity of the survey questions. The survey was then piloted by three pediatric gastroenterology fellows at the lead author's institution, and final implementation of the survey was done with the target study population. Survey available as Supporting Information S2: File S1.

The survey was distributed via REDCap in June 2023 to fellowship graduates registered with NASPGHAN who finished their training between 2018 and 2023 (which included graduates from programs in the United States, Canada, and Mexico). Reminders were sent to non‐respondents during July and August 2023. Alternate email addresses were sought for graduates whose email was not functional. A total of six reminders were sent to all potential respondents over 3 months.

Respondents were asked how comfortable they were at the end of fellowship training performing specific tasks with options including “Yes, for all cases,” “Yes, for all simple cases but would need advice for complex cases,” “Yes, although might talk indirectly with colleague on simple cases,” “No, would need a colleague in the room for direct support,” and “No, not at all.” Responses were dichotomized to affirmative if they would not require immediate support from a colleague and to negative if the respondent was uncomfortable without direct support. Responses to these skill‐based questions were compared to responses to the same questions included in a previous Training Committee survey that was conducted in 2015 among fellows who graduated from 2011 to 2015. Program size was categorized as small (10 faculty or less), medium (11–20 faculty), and large (>20 faculty). Data regarding attendance at fellow conference was obtained from internal records of NASPGHAN.

Statistical analyses were performed using chi‐square testing for survey results in Graphpad Prism 10.2.2. Linear regression was used to test for trends in time. The statistical significance threshold was set at *p*‐value < 0.05.

### 2.1 Ethics statement

This study was deemed to be not human subjects research by the MassGeneralBrigham Institutional Review Board as it was not collecting identifiable information.

## 3 RESULTS

### 3.1 Fellow respondent demographics

A total of 140 responses were received from 676 eligible subjects (21% response rate). Respondents were distributed across all graduation years with the highest subset coming from the Class of 2023 (36% of respondents). Following graduation from pediatric gastroenterology fellowship, 20% of (28/139) respondents completed a 4th year of training. Advanced training in transplant hepatology was the most common 4th year fellowship (10% of all respondents, 14/139) with respondents also indicating training in inflammatory bowel disease (4%, 6/139), motility (3%, 4/139), advanced endoscopy (1%, 2/139), advanced nutrition (1%, 1/139), and research (1%, 1/139). Of those who pursued a 4th year of training, 64% reported their current clinical work effort was >50% in that specific field. Respondents were split among large‐sized programs (36%, 50/140), medium‐sized programs (32%, 45/140), and small‐sized programs (32%, 45/140). Most respondents accepted a job at an academic institution following the end of their training (78%, 103/133). Further description of respondents is included in Table 1 (2023 survey) and Table 2 (2015 survey).

**Table 1:** 2023 survey respondent description.

	*N* = 140
Graduation year	
2018	8% (11/135)
2019	13% (17/135)
2020	12% (16/135)
2021	16% (22/135)
2022	15% (20/135)
2023	36% (49/135)
Size of program (total # of fellows)	
3–5 fellows	25% (35/139)
6–8 fellows	42% (58/139)
9+ fellows	33% (46/139)
Size of faculty (# clinical full time equivalents)	
Small faculty size (1–10 faculty)	32% (45/140)
1–5 faculty	4% (5/140)
6–10 faculty	29% (40/140)
Medium faculty size (11–20 faculty)	32% (45/140)
Large faculty size (>20 faculty)	36% (50/140)
Advanced training	
None	80% (111/139)
Transplant hepatology	10% (14/139)
Inflammatory bowel disease	4% (6/139)
Motility	3% (4/139)
Advanced endoscopy	1% (2/139)
Advanced nutrition	1% (1/139)
Research	1% (1/139)
Type of first job postfellowship	
Academic institution	77% (103/133)
Hospital‐based practice	17% (22/133)
Private practice	5% (7/133)
Industry	1% (1/133)
Reported impact of COVID‐19 pandemic	
Severe impact	8% (11/139)
Moderate impact	52% (72/139)
Minimal impact	18% (25/139)
N/A	22% (31/139)

**Table 2:** 2015 survey respondent description.

	*N* = 57
Gender (male)	57% (32/56)
Number of fellows in fellowship	
1–4 fellows	32% (18/57)
5–9 fellows	49% (28/57)
10–15 fellows	19% (11/57)
What is your current practice/institution?	
Large academic (6+ fellows)	56% (32/57)
Moderate academic (3–4 fellows)	23% (13/57)
Small academic (no fellows)	14% (8/57)
Hospital‐based practice	5% (3/57)
Private practice	2% (1/57)
What year did you graduate?	
2014	23% (13/56)
2013	9% (5/56)
2012	21% (12/56)
2011	20% (11/56)
2010	16% (9/56)
Other	11% (6/56)

#### 3.1.1 Confidence In managing specific disease processes

Respondents reported high levels of confidence (>95% of respondents) in independently managing most common GI tasks including outpatient and inpatient GI cases (100%), inflammatory bowel disease (100%), celiac disease (100%), enteral nutrition assessment (99%), chronic pancreatic disease (97%), and outpatient nontransplant hepatology (97%) with slightly lower rates of confidence for motility cases (92%) and total parental nutrition (90%). Lower overall confidence levels were reported for pre‐transplant liver management (76%) and posttransplant liver management (72%). 2015 data can be seen in Figure S1.

#### 3.1.2 Confidence in performing procedures

Overall confidence in performing common GI procedures independently was high for diagnostic esophagogastroduodenoscopy (100%), diagnostic colonoscopy (99%), and endoscopic removal of foreign bodies (100%). Respondents reported lower confidence for therapeutic procedural skills including endoscopic treatment of upper GI bleeding (76%), intestinal stricture dilation (62%), esophageal variceal banding (60%), and esophageal variceal sclerotherapy (43%). Other procedures with lower confidence levels included rectal suction biopsy (41%), PEG placement (34%), percutaneous liver biopsy (28%), and intestinal ultrasound (4%). Respondents from small‐sized programs reported higher rates of confidence with PEG placement (51%) compared to respondents from medium‐sized programs (24%, *p* = 0.02) and large‐sized programs (27%, *p* = 0.02) as well as percutaneous liver biopsy (47%) compared to medium‐sized programs (18%, *p* = 0.006) and large‐sized programs (20%, *p* = 0.008) (Table 3). Although the confidence level of some procedures remained very consistent across time from 2018 to 2023 (e.g., diagnostic esophagoduodenoscopy and colonoscopy), trends were noted in decreasing confidence for percutaneous liver biopsy (37% over 2018–2020 vs. 22% over 2021–2023, *p* = 0.03) and improving confidence for polypectomy (79% over 2018–2020 vs. 92% over 2021–2023, *p* = 0.02).

**Table 3:** Confidence level of select procedures by faculty size.

	Small size (*n* = 45)	Medium size (*n* = 45)	*p*‐Value	Large size (*n* = 50)	*p*‐Value
Upper gastrointestinal bleeding management	87% (39/45)	76% (34/45)	0.28	68% (34/50)	0.05
Intestinal stricture dilation	68% (30/44)	56% (25/45)	0.28	62% (31/50)	0.67
Variceal banding	71% (32/45)	60% (27/45)	0.38	50% (25/50)	0.04
Variceal sclerotherapy	51% (23/45)	44% (20/45)	0.67	35% (17/49)	0.14
Rectal suction biopsy	34% (15/44)	45% (20/44)	0.38	44% (22/50)	0.4
PEG placement	51% (23/45)	24% (11/45)	0.02	27% (13/49)	0.02
Percutaneous liver biopsy	47% (21/45)	18% (8/44)	0.006	20% (10/50)	0.008

*Note*: Small programs defined as 10 clinical faculty or less, medium‐sized programs as 11–20 faculty and large‐size programs defined as >20 clinical faculty. Abbreviation: PEG, percutaneous endoscopic gastrostomy.

The COVID‐19 pandemic occurred during the training period of 79% (110/140) of respondents with 60% of total respondents (84/140) reporting a moderate to severe impact on their training. Respondents who reported moderate to severe impact from the COVID‐19 pandemic were less likely to feel confident managing upper GI bleeding cases (70% vs. 86%, *p* = 0.042). Confidence levels were similar between those reporting moderate‐severe impact of the COVID‐19 pandemic compared to those with minimal to no difference in performing rectal suction biopsy (35% vs. 52%, *p* = 0.05), intestinal stricture dilation (56% vs. 71%, *p* = 0.11), variceal banding of esophageal varices (55% vs. 68%, *p* = 0.16), and esophageal variceal sclerotherapy (56% vs. 71%, *p* = 0.11) (Table 4).

**Table 4:** Confidence levels of respondents based on COVID‐19 impact on training.

	Moderate–severe COVID impact confidence	Minimal–no COVID impact confidence	*p*‐Value
Preliver transplant evaluation	74% (61/83)	79% (44/56)	0.55
Postliver transplant evaluation	71% (58/82)	75% (42/56)	0.70
Motility cases	92% (76/83)	93% (52/56)	1.0
Percutaneous liver biopsy	23% (19/84)	36% (20/55)	0.09
PEG placement	31% (26/84)	38% (21/55)	0.46
Rectal suction biopsy	35% (29/84)	52% (28/54)	0.052
Upper gastrointestinal bleeding	70% (59/84)	86% (48/56)	0.042
Stricture dilation	56% (47/84)	71% (39/55)	0.11
Variceal banding	55% (46/84)	68% (38/56)	0.16
Variceal sclerotherapy	56% (47/84)	71% (39/55)	0.11

*Note*: *p*‐Values are specific to comparison with small‐sized programs.

Abbreviation: PEG, percutaneous endoscopic gastrostomy.

#### 3.1.3 Confidence in conducting research and applying for grants

Respondents felt less comfortable with conducting basic or translational research (48%, 67/139) compared to clinical research (85%, 119/140, *p* = 0.0001) and quality improvement research (84%, 117/139, *p* = 0.0001). The confidence of respondents from medium‐sized programs to conduct clinical research was lower than that of respondents from large‐sized programs (76% vs. 94%, *p* = 0.018) although respondents from small‐sized programs had similar confidence to those from large‐sized programs (84% vs. 94%, *p* = 0.18). Respondents from large‐sized programs had similar confidence to respondents from medium‐sized and small‐sized programs in conducting basic or translational research (54% vs. 47%, *p* = 0.54; 54% vs. 43%, *p* = 0.31) as well as in conducting quality improvement research (86% vs. 91%, *p* = 0.53; 86% vs. 75%, *p* = 0.2).

**Figure 1:**
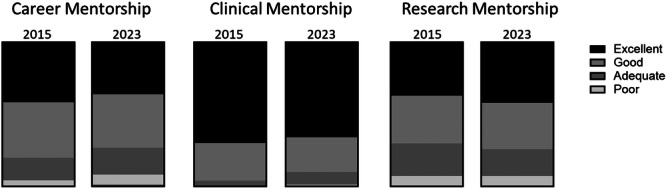
Reported satisfaction with mentorship in 2015 and 2023 NASPGHAN postgraduate surveys. NASPGHAN, North American Society for Pediatric Gastroenterology, Hepatology, and Nutrition.

Overall reported rates of adequate or better mentorship were 99% for clinical mentorship, 91% for career mentorship, and 92% for research mentorship (Table 5, Figure 1). Respondents reported higher rates of outstanding clinical mentorship than career mentorship or research mentorship (66% vs. 36% vs. 42%, *p* = 0.0001). Reported rates of outstanding clinical mentorship and outstanding career mentorship were similar across all program sizes (Table 6). Respondents from large‐sized fellowship programs reported higher rates of outstanding research mentorship compared to respondents from small‐sized programs (59% vs. 22%, *p* = 0.0004).

**Table 5:** Satisfaction levels of mentorship.

	Clinical	Career	Research
Outstanding	66% (92/140)	36% (50/140)	42% (58/139)
Good	24% (34/140)	37% (52/140)	32% (44/139)
Adequate	9% (12/140)	19% (26/140)	19% (26/139)
Poor	1% (2/140)	7% (10/140)	7% (10/139)
Minimal	0% (0/140)	1% (2/140)	1% (1/139)

**Table 6:** Satisfaction levels of mentorship by program size.

	Large‐sized programs (%)	Medium‐sized programs (%)	*p*‐Value large versus medium	Small‐sized programs (%)	*p*‐Value large versus small
Clinical mentorship	70	64	0.66	62	0.52
Career mentorship	48	29	0.06	29	0.06
Research mentorship	59	42	0.15	22	0.0004

A majority of respondents (58%, 80/139) reported that their research mentor did not have R‐ or K‐level funding while 29% (40/139) had a mentor with R funding and 14% (19/139) had a mentor with K funding. Respondents from small‐sized programs were less likely to have an R‐ or K‐funded research mentor than those from large‐sized programs (18% vs. 58%, *p* < 0.0001) and medium‐sized programs (18% vs. 49%, *p* = 0.0033). Outstanding research mentorship was reported more often with R‐funded mentors (68%) and K‐funded mentors (63%) compared to mentors without R or K funding (24%, *p* = 0.0001 and *p* = 0.0009, respectively). Similarly, respondents felt more comfortable writing a research grant if their mentor was R‐ or K‐funded (54% vs. 28%, *p* = 0.002).

#### 3.1.4 Confidence in educational and professional tasks

Respondents reported adequate preparedness by their program for communicating with families (100%), communicating with other physicians (99%), teaching trainees (99%), using the electronic health record (97%), leading a team (91%), and giving feedback to trainees (86%). Most respondents (75%) felt adequately educated by their program on health care disparities in pediatric gastroenterology with similar rates reported by respondents from small‐sized programs (69%) compared to those from medium‐sized programs (73%, *p* = 0.82) and large‐sized programs (82%, *p* = 0.23). Low rates of preparedness were reported for outpatient billing (57%), work‐life balance (55%), selecting a job and negotiating contracts (54%), writing grants (40%), and doing advocacy work (39%). Confidence in writing research grants was similar between those who ultimately took their first posttraining position at an academic institution (41%, 42/103) and those whose first posttraining position was not at an academic institution (27%, 8/30, *p* = 0.20).

For job search related tasks, 74% of respondents felt prepared for job hunting and interviewing and 54% felt prepared for selecting a job and negotiating contracts (with 52% feeling prepared for both tasks). Of note, the resources provided at the NASPGHAN Fellows Conferences were cited by 78% of respondents feeling prepared for the job hunt and interviewing while 66% of respondents that felt prepared for negotiating job contracts and selecting a job cited the NASPGHAN Fellows Conference as being helpful in their process. Compared to the 2015 postgraduate survey, respondents in the current survey reported higher preparedness for billing (57% vs. 26%, *p* = 0.0001), higher preparedness for job hunting and negotiating contracts (52% vs. 30%, *p* = 0.005), and lower preparedness for advocacy work (39% vs. 58%, *p* = 0.007) (Table 7).

**Table 7:** 2023 survey data versus 2025 survey data.

	2023 % (*n*)	2015 % (*n*)	*p*‐Value
Job hunting and negotiation	52% (72/139)	30% (17/57)	0.005
Billing	57% (79/139)	26% (15/57)	0.0001
Advocacy work	39% (53/137)	58% (33/57)	0.007

## 4 DISCUSSION

Fellowship training in pediatric gastroenterology requires a wide range of exposures to maximize the acquisition of cognitive and technical skills across numerous medical conditions and procedures. This survey data demonstrates that the vast majority of fellows graduate with the confidence to manage common and major disease processes as well as commonly performed GI procedures. Respondents reported a high level of preparation for verbal and electronic communication with patients, families and team members.

Confidence levels in graduates from pediatric gastroenterology fellowship programs have not been extensively studied. This survey reports higher confidence levels in treating patients with inflammatory bowel disease compared to a prior survey carried out by the Crohn's and Colitis Foundation.^4^ Our data may represent a true increase in confidence over time or may relate to slightly different wording in survey questions. Another key finding in this study is that confidence levels in many training topics remained high despite 60% of respondents reporting a moderate to severe impact from the COVID‐19 pandemic on their training. It is worthwhile to point out that the survey tool relied on responders to define the impact from COVID‐19 on their training rather than using a strict definition of impact so the complexity of the relationship of COVID‐19 may not be fully understood.

Pediatric GI Training Guidelines describe esophagogastroduodenoscopy, endoscopic foreign body retrieval, colonoscopy, polypectomy, and bleeding control procedures as required procedures during training.^5^ The confidence level in these procedures remained high. The confidence in performing bleeding control procedures decreased during the COVID‐19 pandemic which is not surprising given that previous data demonstrates that it is uncommon for trainees to get the recommended number of procedures during fellowship.^6^ Confidence levels for optional procedures as defined by Pediatric GI Training Guidelines such as percutaneous liver biopsy, PEG placement, and rectal suction biopsies decreased which likely represents an evolving trend at many institutions where other subspecialties perform most of these procedures. It is not clear if the relative low confidence levels for performing rectal suction biopsies was due to other specialties performing rectal suction biopsies or whether their performance has declined slightly with the use of other diagnostic testing (such as anorectal manometry) in the diagnostic algorithm of Hirschsprung's disease. Although the role of point‐of‐care intestinal ultrasound is rapidly evolving, this survey data demonstrates that training of fellows has largely not yet begun.

These data also demonstrate important trends in other aspects of training. Education on health care disparities in pediatric gastroenterology was found to be adequate by 75% of fellow graduates with similar rates reported between large and small programs. However, it does demonstrate that additional work is needed to adequately prepare fellows with regard to healthcare disparities. On a related note, this survey identifies the need to improve training in advocacy with less than half of respondents feeling adequately prepared. With growing recognition of the role of the pediatric gastroenterologist to advocate for change, the decreased rates of preparedness for advocacy work in this survey should be a call to NASPGHAN and individual programs to augment exposure and training in advocacy.

**Figure 2:**
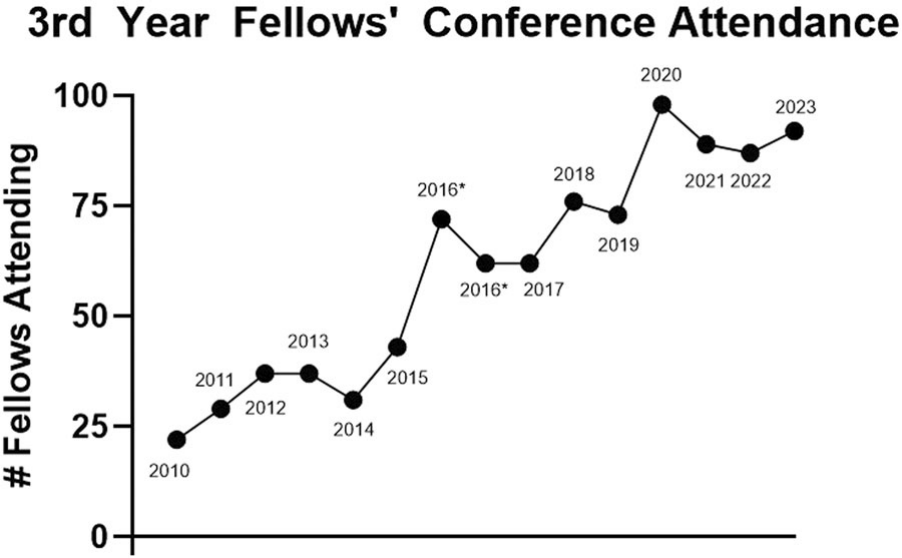
Attendance at NASPGHAN 3rd year fellow conference by year. Two conferences occurred during 2016 as timing of conference moved from spring of 3rd year to fall of 3rd year. 2020 conference was held virtually. NASPGHAN, North American Society for Pediatric Gastroenterology, Hepatology, and Nutrition.

The process of interviewing for the first posttraining job in pediatric gastroenterology became more complex during the COVID‐19 pandemic.^3^ This survey demonstrates an increased level of self‐reported preparedness for the job interview process compared to the 2015 survey. These changes may reflect changes made to NASPGHAN's career development programming available to all fellows in North America. In 2016, NASPGHAN's 3rd year fellows conference shifted in focus from research presentation to general career development with a focus on the job search, negotiation, and transition to life as faculty. After that transition, absolute numbers of fellows attending the 3rd year fellows conference increased as did the percentage of eligible fellows attending (with 2012–2015 attendance ranging from 37% to 40% of the 3rd year class and rose to 63% for the second conference in 2016) (Figure 2). These data provide evidence that NASPGHAN Fellows Conferences are a commonly shared opportunity for trainees in pediatric gastroenterology to have such needs met.

Mentorship during training remains a critical factor in career development, and respondents reported varying satisfaction across different types of mentorship. Satisfaction with clinical mentorship was highest among the domains tested and was similar across different sizes of training programs. Career mentorship showed lower satisfaction from respondents and although there was no statistical difference between the size of the programs, there is a trend that suggests small‐sized and medium‐sized programs should invest further effort into career mentorship. Reported rates of outstanding research mentorship were higher at large‐sized programs than small‐sized programs as well as for respondents with R‐ or K‐funded mentors. NASPGHAN has had programs to facilitate research mentorship between trainees and faculty at other institutions, although it is not clear whether fellows select research projects based on locally available resources and thus not be in a position to utilize these opportunities. Importantly, the data demonstrate that individual programs should ensure their trainees are aware of mentorship resources both at the local institution level and at the NASPGHAN level.

The data obtained in this survey does contain several limitations. The relatively low response rate from graduates does have a potential impact on generalizability although this response rate is similar to recent NASPGHAN survey response rates.^3^, ^7^ Future postgraduate surveys may benefit from a longitudinal approach to recruit responders from the graduating class on a yearly basis given the higher participation rate amongst immediate graduates. Given that the target population of this survey includes fellowship program graduates that train in the United States, Canada, and Mexico, the impact of the Accreditation Council for Graduate Medical Education (ACGME) requirements as well as differential duration of program training may also influence these results. A final consideration of the interpretation of these results is that confidence in a task or skill may not perfectly correlate with competency in that item. Training programs are tasked to administer and report competency‐based assessments through ACGME milestones and EPAs. Future research may shed light on the correlation between self‐assessed confidence and program‐assessed competency and entrustment.

## 5 CONCLUSION

Overall, this survey describes high levels of confidence among pediatric gastroenterology fellowship graduates in completing many of the core activities of the field. It demonstrates improvement in some areas that have recently been a focus of NASPGHAN (through changes in the content presented during fellows conferences) and the pediatric gastroenterology community (i.e., education in healthcare disparities and preparedness for activities related to fellow‐to‐faculty transition) although additional work is clearly needed in these areas. Finally, it does identify some areas that require additional resources, such as preparing our graduating trainees for advocacy work.

## CONFLICT OF INTEREST STATEMENT

The authors declare no conflict of interest.

## Supporting information

Supporting Figure 1 Legend: Reported Confidence Levels (%) in 2015 NASPGHAN Postgraduate Survey.

Supplemental Data File: List of Survey Questions; Abbreviations: Gastrointestinal (GI), Inflammatory Bowel Disease (IBD), Total Parenteral Nutrition (TPN), Esophagogastroduodenoscopy (EGD), North American Society for Pediatric Gastroenterology, Hepatology, and Nutrition (NASPGHAN), National Institute of Health (NIH), History and Physical (H&P), Emergency Department (ED), Nurse (RN).
