# Supplementary figures and images for: Posttraining survey of recent pediatric gastroenterology fellowship graduates
Source: JPGN Rep. 2025 Jun 24;6(4):334–41. doi: 10.1002/jpr3.70050 (PMC12611581; doi:10.1002/jpr3.70050)

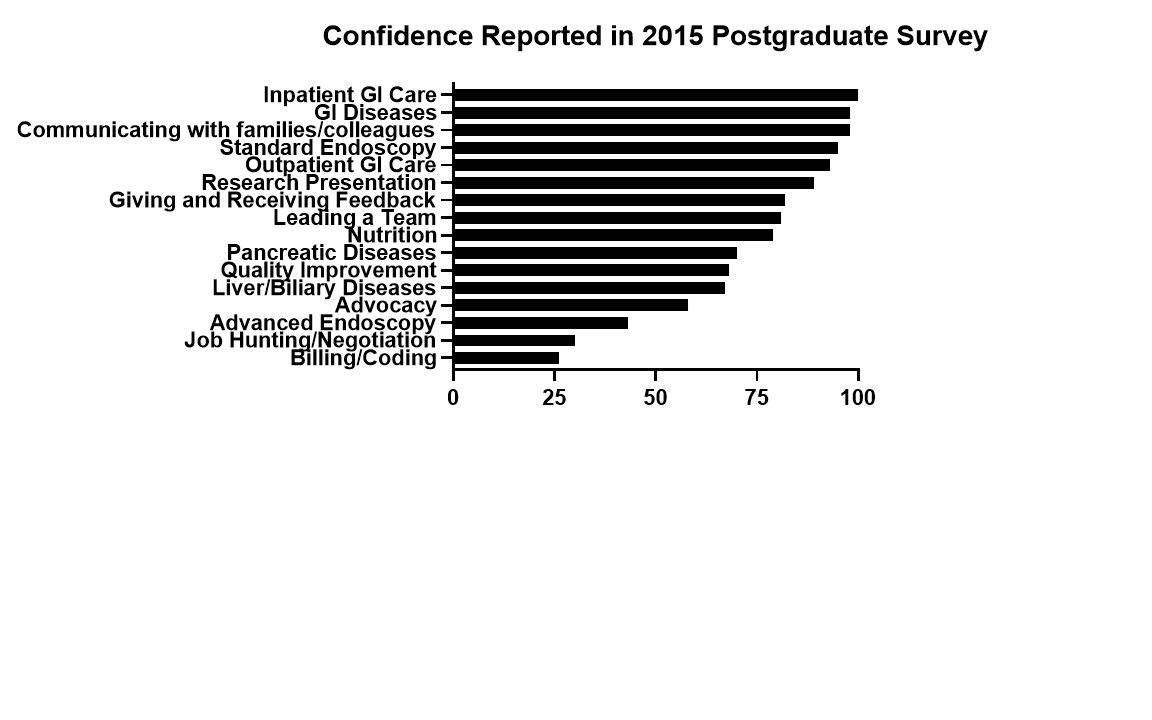

Supplement: Supplementary file 1 — Supporting Figure 1 Legend: Reported Confidence Levels (%) in 2015 NASPGHAN Postgraduate Survey. [file JPR3-6-334-s002.tif]
