# Supplementary material for: Posttraining survey of recent pediatric gastroenterology fellowship graduates
Source: JPGN Rep. 2025 Jun 24;6(4):334–41. doi: 10.1002/jpr3.70050 (PMC12611581; doi:10.1002/jpr3.70050)
Supplement: Supplementary file 2 — Supplemental Data File: List of Survey Questions; Abbreviations: Gastrointestinal (GI), Inflammatory Bowel Disease (IBD), Total Parenteral Nutrition (TPN), Esophagogastroduodenoscopy (EGD), North American Society for Pediatric Gastroenterology, Hepatology, and Nutrition (NASPGHAN), National Institute of Health (NIH), History and Physical (H&P), Emergency Department (ED), Nurse (RN). [file JPR3-6-334-s001.docx]

1 **How prepared did you feel AT THE END OF YOUR FELLOWSHIP for the following:**

|  | No, not at all | No, I’d need another attending directly in the room | Yes, although I might talk indirectly with another attending for simple cases | Yes, for all simple cases (but would need advice for complex cases) | Yes for all cases |
| --- | --- | --- | --- | --- | --- |
| Outpatient General GI management |  |  |  |  |  |
| Inpatient General GI Management |  |  |  |  |  |
| Outpatient IBD Management |  |  |  |  |  |
| Outpatient Celiac Management |  |  |  |  |  |
| Outpatient Motilty/Neurogastroenterology Management |  |  |  |  |  |
| Outpatient Non-Transplant Hepatobiliary Disease Management |  |  |  |  |  |
| Pre-Liver Transplant Evaluation |  |  |  |  |  |
| Post-Liver Transplant Management |  |  |  |  |  |
| Outpatient Pancreatic Disease Management |  |  |  |  |  |
| Enteral Nutrition Assessment and Management |  |  |  |  |  |
| TPN Management |  |  |  |  |  |

2 **How comfortable (AT THE END OF YOUR FELLOWSHIP) were you for the following:**

|  | No, not at all | No, I’d need another attending directly in the room | Yes, although I might talk indirectly with another attending for simple cases | Yes, for all simple cases (but would need advice for complex cases) | Yes for all cases |
| --- | --- | --- | --- | --- | --- |
| Performing Diagnostic EGD |  |  |  |  |  |
| Performing Diagnostic Colonoscopy |  |  |  |  |  |
| Performing Endoscopic Foreign Body Removal |  |  |  |  |  |
| Performing Rectal Suction Biopsy |  |  |  |  |  |
| Performing Polypectomy |  |  |  |  |  |
| Performing Endoscopic Treatment of Upper GI Bleeding (Clips, Cautery, Hemospray) |  |  |  |  |  |
| Performing Variceal Banding |  |  |  |  |  |
| Performing Variceal Sclerotherapy |  |  |  |  |  |
| Performing Percutaneous Liver Biopsy |  |  |  |  |  |
| Placing PEG Tubes |  |  |  |  |  |
| Performing Esophageal/Intestinal Stricture Dilation |  |  |  |  |  |
| Performing Bedside Intestinal Ultrasound |  |  |  |  |  |

3. **Did you feel adequately prepared AT THE END OF FELLOWSHIP for the following research domains:**

|  | Yes | No |
| --- | --- | --- |
| Conducting Basic/Translational Research |  |  |
| Conducting Clinical Research |  |  |
| Conducting Quality Improvement Research |  |  |

4a Preparation for Various Tasks

|  | Yes | No |
| --- | --- | --- |
| **Did you feel adequately educated on health care disparities?** |  |  |
| **Did you feel adequately prepared after fellowship for billing for visits?** |  |  |
| **Did you feel adequately prepared after fellowship for job hunting and interviewing?** |  |  |
| **Did you feel adequately prepared for selecting a job and negotiating contracts?** |  |  |
| **Did you feel adequately prepared for conducting telehealth visits?** |  |  |
| **Did you feel adequately prepared to give oral presentations at national conferences?** |  |  |
| **Did you feel adequately prepared to make posters for conferences?** |  |  |
| **Did you feel adequately prepared to write grants?** |  |  |
| **Did you feel adequately prepared to lead a team?** |  |  |
| **Did you feel adequately prepared to teach trainees?** |  |  |
| **Did you feel adequately prepared to give feedback to trainees?** |  |  |
| **Did you feel adequately prepared to engage in quality improvement work?** |  |  |
| **Did you feel adequately prepared to communicate with patients and families?** |  |  |
| **Did you feel adequately prepared to communicate with other physicians?** |  |  |
| **Did you feel adequately trained to effectively use the electronic health record?** |  |  |
| **Did you feel adequately prepared to do advocacy work?** |  |  |
| **Did you feel adequately trained to deal with Work-Life balance?** |  |  |

4b: For any 4a question with “Yes” answer, secondary question:

What resources did you use to help prepare yourself for this?
- Outpatient Clinic

- Inpatient rotations
- My program’s didactics
- Primary research mentor
- Other mentors in my program
- NASPGHAN fellows conferences
- Other
- Did not receive training

4c: What professional resources have you found most helpful since leaving fellowship? (Open response)

4d: What additional resources for fellows or early career faculty could be developed by NASPGHAN to address gaps in your training:

- Grant writing
- Manuscript writing
- Clinical program development
- Quality improvement
- Leadership training
- Teaching trainees
- Time management skills
- Work-life integration
- Academic promotion
- Business management
- Wellness/Resilience
- Other (Open response)

5a **Rate the Mentorship that you received during fellowship**

|  | Minimal | Poor | Adequate | Good | Outstanding |
| --- | --- | --- | --- | --- | --- |
| Career Mentorship |  |  |  |  |  |
| Clinical Mentorship |  |  |  |  |  |
| Research Mentorship |  |  |  |  |  |

.

5b: Did your primary research mentor have funding:

- R level NIH funding
- K level NIH funding
- Other NIH funding
- Foundation grant funding
- Philanthropic support
- My research mentor did not have grant funding
- I did not have a research mentor

6 What year did you graduate from Pediatric GI fellowship?

7 How many fellows were in your program (counting all years of the program)?

8 How many attending physicians (who perform some amount of clinical care) were in your program that you trained in Pediatric GI (when you were there)?

- 0-5 attendings
- 6-10 attendings
- 11-20 attendings
- >20 attendings

9a Did you complete a 4^th^ year advanced fellowship? Yes/No

9b Which 4^th^ year advanced training fellowship?

- Transplant hepatology
- Motility
- Advanced nutrition
- Inflammatory Bowel Disease
- Advanced Endoscopy
- Other

9c If you completed a 4^th^ year advanced fellowship, are you practicing primarily (>50% of your clinical time) in that area of training? Yes/No

10a Did the COVID-19 pandemic occur during your training Yes/No

10b To what degree did the COVID-19 pandemic affect your fellowship training?

- No Impact
- Minimal impact
- Moderate impact
- Severe impact

11 Did the hospital you trained at perform pediatric liver transplants: Yes/No

12 What type of inpatient notes were you typically required to write (rather than addend resident notes or not write any at all)? Choose all that apply:

- H&P on all patients on primary GI service
- H&P on all consult patients
- Progress notes on patients on primary GI service
- Progress notes on all consult patients
- My program did not require fellows to write their own notes

13 Describe how calls from outpatients were handled on nights/weekends when you were on call during that fellowship (choose all that apply):

- First call for external ED calls
- First call for internal ED calls
- First call for all outpatients
- First call after RN triage system if needed
- Attending took first call on some nights

14 Did you feel significantly sleep deprived during your first year of fellowship?

- Never
- Yes, but only once or twice
- Yes, about once per month
- Yes, at least once a week

15 How much time was available to you for maternity/paternity/family leave for birth/adoption of a child, without using paid time off:

- None
- 2 weeks
- 4 weeks
- 6 weeks
- 8 weeks
- 12 weeks
- Other
- I don’t know

16 What gaps in fellowship training have you identified since graduation?

17 Please select which best describes your first post-fellowship job:

- Academic institution
- Hospital-based practice
- Private practice
- Industry
- Other

18 Was your first post-fellowship job at the institution where you did your fellowship: Yes/NO

19 **Rank the following factors in terms of importance of why you selected your first post-fellowship job (with 1 being most important and 6 being least important)**

|  | 1 | 2 | 3 | 4 | 5 | 6 |
| --- | --- | --- | --- | --- | --- | --- |
| Geographic location |  |  |  |  |  |  |
| Institution/practice quality |  |  |  |  |  |  |
| Salary |  |  |  |  |  |  |
| Specific job definition |  |  |  |  |  |  |
| Personal/Family Considerations |  |  |  |  |  |  |
| Other |  |  |  |  |  |  |

20 Have you left that first post-fellowship job for another job?

21 In general, what has been the most challenging aspect of your life post-fellowship?
